# Hedgehog/GLI signaling in tumor immunity - new therapeutic opportunities and clinical implications

**DOI:** 10.1186/s12964-019-0459-7

**Published:** 2019-12-26

**Authors:** Sandra Grund-Gröschke, Georg Stockmaier, Fritz Aberger

**Affiliations:** Department of Biosciences, Cancer Cluster Salzburg, University of Salzburg, Hellbrunner Strasse, 34, 5020 Salzburg, Austria

**Keywords:** Oncogenic Hedgehog/GLI signaling, Tumor microenvironment, Cancer immunotherapy, Immunosuppression, Immune evasion, Immune checkpoint inhibitors, Chronic inflammation, Combination therapy

## Abstract

Uncontrolled activation of the Hedgehog/Glioma-associated oncogene (HH/GLI) pathway is a potent oncogenic driver signal promoting numerous cancer hallmarks such as proliferation, survival, angiogenesis, metastasis and metabolic rewiring. Several HH pathway inhibitors have already been approved for medical therapy of advanced and metastatic basal cell carcinoma and acute myeloid leukemia with partially impressive therapeutic activity. However, de novo and acquired resistance as well as severe side effects and unexplained lack of therapeutic efficacy are major challenges that urgently call for improved treatment options with more durable responses. The recent breakthroughs in cancer immunotherapy have changed our current understanding of targeted therapy and opened up promising therapeutic opportunities including combinations of selective cancer pathway and immune checkpoint inhibitors. Although HH/GLI signaling has been intensely studied with respect to the classical hallmarks of cancer, its role in the modulation of the anti-tumoral immune response has only become evident in recent studies. These have uncovered HH/GLI regulated immunosuppressive mechanisms such as enhanced regulatory T-cell formation and production of immunosuppressive cytokines. In light of these exciting novel data on oncogenic HH/GLI signaling in immune cross-talk and modulation, we summarize and connect in this review the existing knowledge from different HH-related cancers and chronic inflammatory diseases. This is to provide a basis for the investigation and evaluation of novel treatments combining immunotherapeutic strategies with approved as well as next-generation HH/GLI inhibitors. Further, we also critically discuss recent studies demonstrating a possible negative impact of current HH/GLI pathway inhibitors on the anti-tumoral immune response, which may explain some of the disappointing results of several oncological trials with anti-HH drugs.

Additional file 1Video abstract. (9500 kb)

Video abstract. (9500 kb)

## Background

Since its discovery in the 1980s by Christiane Nüsslein-Vollhard and Eric Wieschaus the Hedgehog/Glioma-associated oncogene (HH/GLI) signaling pathway has been studied in great detail [1]. HH/GLI signaling can orchestrate several central developmental processes, including pattern and limb formation in the embryonic development or cell proliferation and differentiation. In the adult organisms the pathway is mostly inactive but reactivated during tissue homeostasis and regeneration as well as in the process of wound healing by controlling stem cell activation and self-renewal.

Unlike most classical signaling cascades, HH/GLI signaling is actively repressed in the absence of ligand and initiated by binding of HH ligand protein to its receptor and pathway repressor Patched (PTCH1). In addition to mere receptor binding, this step also apparently inactivates the catalytic activity of PTCH1, thereby changing the cholesterol composition within the leaflets of the lipid bilayer of the cell membrane close to the primary cilium, an antenna-like compartment critical for the coordination of HH/GLI signal strength and duration. HH ligand binding relieves the repressive function of PTCH1, thereby allowing the translocation of the G-protein coupled receptor-like protein Smoothened (SMO) into the primary cilium, where its activation results in the conversion of transcriptionally repressive GLI zinc-finger transcription factors into transcriptional activator forms [2–11].

While the exquisite and precise quantitative control of HH/GLI signaling in space and time is mandatory for normal development and health of mammals, irreversible and uncontrolled activation of the HH/GLI pathway is detrimental and has been shown to cause or contribute to the development of a variety of cancer entities. For instance, HH/GLI represents a key molecular driver signal in basal cell carcinoma (BCC), medulloblastoma (MB) and rhabdomyosarcoma and has been implicated in the malignant progression of for instance gastrointestinal, pancreatic, ovarian, breast, prostate and lung cancers, melanoma, glioma, and several leukemia including chronic lymphocytic leukemia (CLL), chronic myeloid leukemia (CML), diffuse large B-cell lymphoma (DLBCL) and acute myeloid leukemia (AML) (for reviews see [2, 4, 12–22] and references therein).

In light of the critical role of HH/GLI in various malignancies and oncogenic processes, several clinically suitable HH pathway inhibitors have been successfully developed. This is reflected by the approval of the first SMO inhibitor vismodegib (GDC-0449, ERIVEDGE™) in 2012 for the treatment of locally advanced and metastatic BCC [23–27], followed by the approval of sonidegib (LDE225, ODOMZO™) after having shown therapeutic efficacy in BCC patients [28–31]. Only recently, the SMO antagonist glasdegib (PF-04449913, DAURISMO™) has been approved in combination with low-dose chemotherapy for the treatment of acute myeloid leukemia patients after clinical studies have shown nearly a doubling of the overall survival of AML patients if glasdegib is included in the low-dose chemotherapy regimen [32, 33]. Furthermore, the clinically approved chemotherapeutic agent arsenic trioxide (ATO) (TRISENOX™) has been identified as potent inhibitor of GLI activity, adding another promising compound to the growing drug armamentarium against HH-driven cancers [34, 35].

Despite the impressive therapeutic efficacy of HH pathway inhibitors, de novo and acquired drug resistance as well as severe side effects are major limitations to the successful use of SMO antagonists [29, 36–38]. Of note, 50% of the BCC patients that show resistance to the SMO inhibitors express mutant SMO variants and show maintained high-level HH/GLI pathway activity. Mutations occur either directly in the ligand binding pocket (LBP) of SMO or outside the LBP in pivotal regions of the transmembrane-helices that ensure receptor auto-inhibition. Further resistance mechanisms comprise GLI2 gene amplifications, loss of the GLI repressor Suppressor of Fused (SUFU), or a signaling shift towards protein kinase C (PKC), phosphatidyl inositol-3 kinase (PI3K) and/or mitogen activated protein kinase (MAPK) activity [39–44].

Such therapeutic challenges call for improved treatment strategies for the patients´ benefit. The recent breakthroughs in cancer immunology and immunotherapy have highlighted the necessity for a precise understanding of the immune-modulatory function of oncogenic signaling pathways and their actual role in tumor immunity. Such precise and context-dependent knowledge is mandatory for the development of rational combination treatments targeting for instance oncogenic and immunosuppressive signals. Along the same line, it is equally important to understand the role of HH/GLI in tumor immunity in both the tumor itself as well as in the immune microenvironment of the cancer to guide and select the most efficient drug combination with more durable responses and increased response rates.

As for HH/GLI signaling, recent studies have linked HH/GLI pathway activation with concomitant anti-inflammatory signals [45, 46] and revealed a significant downregulation of the pathway in a set of chronic inflammatory diseases such as inflammatory bowel disease [47], colitis [48, 49] and *Helicobacter pylori* associated gastric inflammation [21, 50]. Notably, there is also increasing evidence, showing that oncogenic HH/GLI signaling regulates immunosuppressive mechanisms such as enhanced regulatory T-cell (Treg) formation and production of immunosuppressive cytokines, which can open new avenues for combination treatments and immunotherapy [49, 51–56].

In light of these recent insights, we here summarize and reconcile the existing knowledge from different HH/GLI-related cancers and chronic inflammatory diseases and discuss the relevance of HH/GLI signaling in modulating the immune response, which should provide a basis for the future evaluation of novel treatment options and may also help explaining the failure of HH pathway inhibitors in several clinical trials [57].

### HH signaling and tumor immunity

**Fig. 1:**
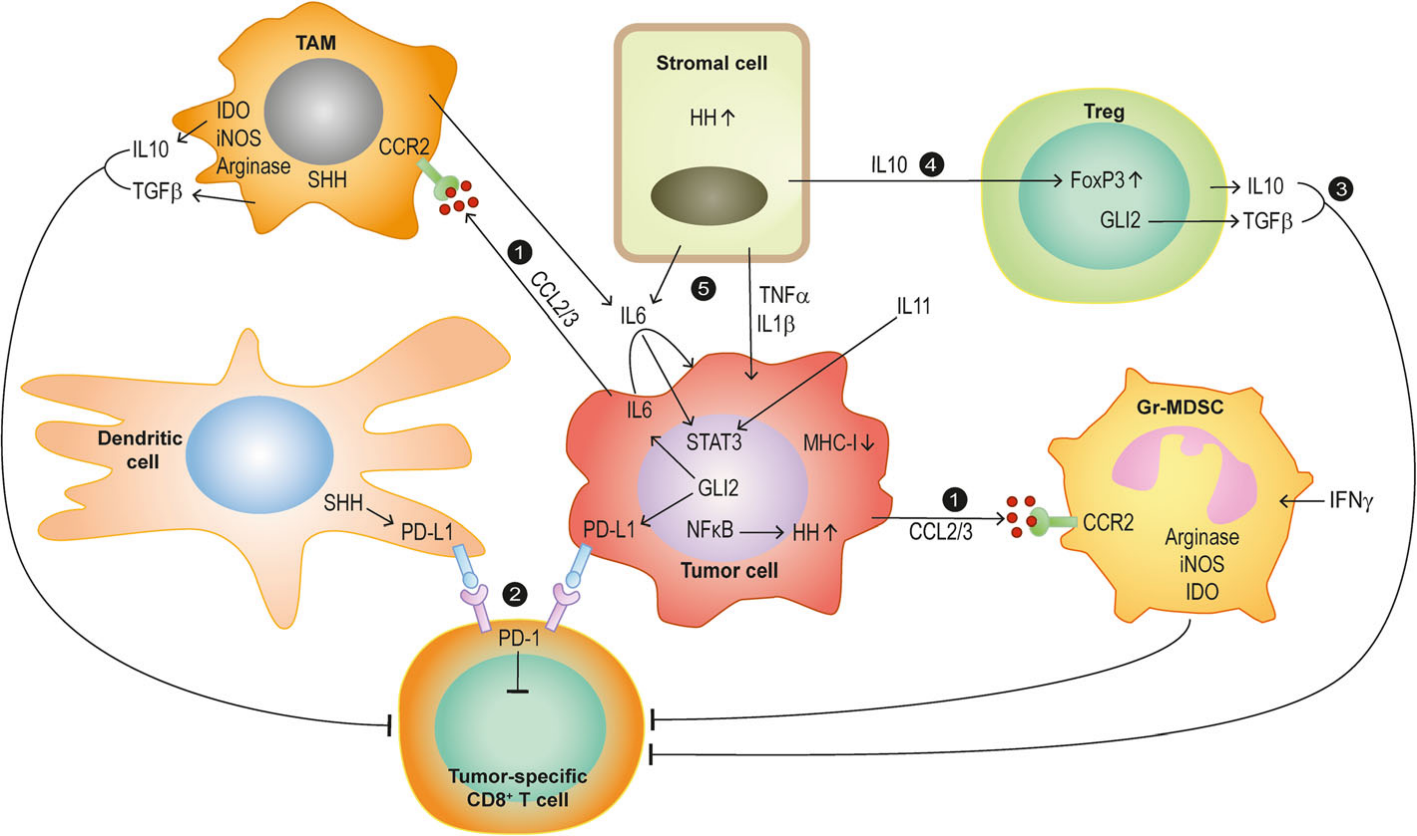
Mechanisms of immune modulation by HH/GLI signaling in cancer and inflammation. 1) Cancer cells release CCL2/3 in response to oncogenic HH/GLI signaling, thereby recruiting TAMs and immunosuppressive MDSCs. 2) HH/GLI-induced PD-L1 expression in cancer and dendritic cells inhibits tumor specific cytotoxic T-cells via binding to PD-1. 3) GLI2 drives production of immunosuppressive cytokines and growth factors (IL10 and TGFβ), which results in the inactivation of tumor specific CD8^+^ T-cells. 4) HH/GLI-induced IL10 from stromal cells promotes FoxP3 expression in regulatory T-cells. 5) Pro-inflammatory signals such as IL6/STAT3 interact with HH/GLI signaling; HH/GLI-induced autocrine IL6 signaling and/or pro-inflammatory IL6 from TAM and stromal cells activate STAT3 signaling in cancer cells, thereby promoting malignant growth

The adaptive as well as innate immune system forms a highly proficient immune surveillance machinery that recognizes and destroys genetically altered cells to prevent the development of malignant diseases. Cancer development driven by genetic and epigenetic evolution and clonal selection, therefore, involves a plethora of molecular mechanisms that eventually lead to the suppression of the anti-tumoral response and immune evasion of malignant cells, respectively [58]. Notably, the administration of for instance immune checkpoint inhibitors that efficiently re-instate the anti-tumoral immune response have shown unprecedented therapeutic efficacy in several metastatic diseases [59–61], suggesting that rational combination treatments targeting oncogenic HH/GLI and immunosuppressive mechanisms may synergistically improve the efficacy and durability of the therapeutic response of patients suffering from HH/GLI-associated cancers. In the following chapter we summarize recent findings about the implication of HH/GLI signaling in the context of immunosuppression and immune evasion (summarized in Fig. 1).

Mutational activation of HH/GLI signaling plays a causal role in the development and growth of BCC. Intriguingly, systematic genome sequencing of several hundreds of sporadic human BCC revealed a surprisingly high mutational burden with an average of 65 mutations per megabase [62]. Although these sequencing data have not yet been analyzed with respect to the immunogenicity of the mutations, it is highly likely that BCC express tumor-specific neoantigens rendering BCC lesions immunogenic. We, therefore, hypothesize that HH/GLI signaling – in addition to tumor-intrinsic proliferative and pro-survival cues – also induces an immunosuppressive microenvironment to hamper an effective anti-tumoral immune response.

First evidence for such immunosuppressive mechanisms in BCC came from studies of murine BCC models showing that transforming growth factor beta (TGFβ) secreted by oncogenic SMO-expressing keratinocytes is able to reduce the number of effector lymphocytes in the tumor tissue. In addition, TGFβ signaling in bone marrow cells of BCC mice appears to support tumor growth by recruiting immunosuppressive myeloid derived suppressor cells (MDSC) to BCC lesions in a C-C motif chemokine ligand 2 (CCL2) dependent manner (Fig. 1). In agreement, pharmacologic inhibition of the CCL2 receptor expressed by MDSCs not only interfered with the recruitment of these cells but also reduced tumor growth. However, the detailed anti-tumoral mechanisms in response to CCL2 receptor inhibition remain elusive [53, 63].

Further evidence that HH signaling induces immunosuppressive mechanisms such as MDSC recruitment and M2 polarization of macrophages was provided by the analysis of an immunocompetent breast cancer xenograft mouse model. Treatment of engrafted mice with the SMO inhibitor vismodegib reduced immunosuppressive immune cell populations such as MDSCs, M2 macrophages and Treg cells in the tumor lesions, while it increased the number of cytotoxic CD8^+^ T-cells and M1 macrophages, resulting in less metastasis. Notably, macrophage depletion in combination with HH pathway inhibition further improved the therapeutic effect of HH blockade alone [64].

Analysis of human UV-exposed facial BCC revealed that Treg cells accumulate in high amount within intra- and peritumoral regions. This is accompanied by a strong increase of immunosuppressive TGFβ in the peritumoral skin [54]. In this context, it is intriguing to mention that the HH effector and zinc finger transcription factor GLI2 can directly activate the expression of TGFβ in human Treg cells [55] (Fig. 1). This immune modulatory role of HH/GLI signaling in T-cells is further underlined by a study showing that GLI2 can attenuate T-cell activation and function by altering gene expression profiles in T-cells. GLI2 activation results in impaired TCR-induced calcium influx and differential expression of major components of the TCR signaling pathway such as nuclear factor kappa B (NFκB) and activator protein-1 (AP-1) factors [65]. Furthermore, HH/GLI signaling is able to polarize Th2 differentiation of T-cells by inducing interleukin-4 (IL4) production, thereby promoting allergic responses and reducing cytotoxic T-cell function in the context of tumor immunity [66, 67]. In addition to T-cell polarization, activation of HH/GLI in naïve CD4^+^ T-cells in the context of atopic dermatitis development has been shown to induce the differentiation of immunosuppressive Treg cells expressing elevated FOXP3 and TGFβ levels [68] (Fig. 1). Despite convincing evidence for a cell-autonomous role of HH/GLI in Treg formation, it remains unclear of whether T-cell intrinsic activation of HH/GLI also plays an immunosuppressive role in the microenvironment of HH-driven cancers such as BCC.

Further evidence for a role of HH/GLI in Treg formation comes from the analysis of patients infected with *Mycobacterium tuberculosis*. In this study, mycobacteria-infected human DCs upregulated SHH signaling, which in turn was able to induce programmed death ligand 1 (PD-L1) expression (Fig. 1). This resulted in Treg formation and expansion, thereby favoring immune evasion of the pathogen [51]. In addition to this, using human-derived gastric cancer organoids it was demonstrated that GANT-61 could reduce PD-L1 expression and tumor cell proliferation in vitro and in vivo. Of note, treatment with anti-PD-L1 antibodies induced apoptosis of tumor cells derived from GLI2-expressing mouse organoids. The results identify GLI2 as tumor-cell intrinsic regulator of PD-L1 expression in gastric cancer, promoting cancer growth via suppression of anti-tumoral responses [56].

Aside from the effects of HH/GLI on immunosuppression in malignant settings, Sonic HH (SHH)-induced Treg formation can also constrain inflammation driven diseases [49, 52]. For instance, in colitis, activation of HH/GLI signaling dampens the inflammation, thereby preventing inflammatory intestinal damage. In this context Lee et al. showed increased interleukin-10 (Il10) expression by Gli1-positive stromal cells upon chemical HH pathway activation together with an increased number of Treg cells [49] (Fig. 1). A similar mechanism was discovered in a mouse acute pancreatitis model, where autocrine Shh signaling induced Il10 production resulting in reduced inflammation [52]. Notably, inhibition of Hh/Gli signaling worsened the progression of the inflammatory disease and promoted colitis-associated cancer development [49, 52]. It is, therefore, conceivable that pro-inflammatory responses to SMO-targeting contributed to the failure of colon cancer trials, where drug targeting of HH signaling accelerated cancer progression, which forced the termination of the clinical studies.

The immunosuppressive activity of HH/GLI in the intestine is further supported by a study of Westendrop et al. showing that epithelial-derived Indian hedgehog (Ihh) is able to maintain immune tolerance in the intestine. Loss of Ihh from the intestinal epithelium led to increased expression of inflammation-related genes and an influx of immune cells. Mechanistically, Ihh can inhibit the release of the chemokine CXCL12 by fibroblasts and thereby, reduce the recruitment of immune cells. In agreement with its immunosuppressive function, loss of Ihh resulted in increased colitis [69].

In the context of *Helicobacter pylori*-induced gastric inflammation and carcinogenesis, the bacterial infection has been shown to induce SHH signaling via activation of NFκB signaling [70] (Fig. 1). During chronic gastric inflammation, HH/GLI signaling can support polarization of myeloid cells towards granulocytic-MDSCs (GrMDSCs) (Fig. 1) [71, 72]. SHH secreted from parietal cells has been shown to attract Schlafen 4 (SLFN4)-positive myeloid cells from the bone marrow. SLFN4-positive myeloid cells can further polarize by IFNα to GrMDSCs, which then are able to inhibit tumor infiltrating lymphocytes, favoring neoplastic transformation and cancer growth [21, 71, 73]. Interestingly, El-Zaatari et al. provided additional evidence for a critical role of HH/GLI in immune suppression and malignant transformation, since Gli1 deletion in mice prevented both *Helicobacter pylori*-induced expansion of MDSCs and metaplasia [50].

As already mentioned above in the context of HH/GLI-dependent Treg formation, HH/GLI is likely to cause immunosuppression also by inducing the expression of immune checkpoint molecules. For instance, in BCC with tumoral PD-L1 expression, two patients with metastatic disease responded well to αPD-1 single treatment with nivolumab or pembrolizumab [74, 75]. Lipson et al. further analyzed 40 BCC specimens and found PD-L1 expression on tumor cells to be upregulated in 22% of all analyzed samples with an additional 82% of specimen demonstrating PD-L1 expression on tumor infiltrating lymphocytes and associated macrophages (Fig. 1) [76]. Intriguingly, PD-L1 upregulation was also observed in some medulloblastoma patients, were the highest PD-L1 expression was found in a patient with SHH subtype MB [77]. Together with the results of a study with GLI2-expressing PD-L1-positive gastric organoids [56], these data together suggest a direct regulatory function of HH/GLI in the control of immune checkpoint expression.

Finally, another mechanism how cancer cells can evade the immune system is by downregulating MHC-I expression, whereby tumor antigen-specific T-cells are then no longer capable of recognizing and destroying these abnormal cells [58, 78]. Recently, inhibition of the HH pathway in BCC patients with the SMO Inhibitors vismodegib or sonidegib led to increased levels of MHC-I expression on tumor cells together with an increase of CD4 and CD8 positive T-cells in the peri- and intra-tumoral regions. These findings indicate that MHC-I downregulation occurs in HH-driven BCC to evade the recognition and destruction by the immune system [79].

In summary, there is increasing evidence from multiple studies that demonstrate an important and multi-facetted immune-modulatory role of HH/GLI in various inflammatory and malignant settings. Active HH/GLI signaling can induce an immunosuppressive microenvironment via multiple routes, including the activation of immunosuppressive cytokines, upregulation of immune checkpoints, or expansion and chemotactic recruitment of immunosuppressive cells including Treg and MDSCs. The immune-modulatory activity of HH/GLI in cancer settings thus opens up new therapeutic avenues for future treatment strategies of HH/GLI associated cancers.

### Oncogenic HH/GLI signaling and inflammation

The immune system plays a decisive and at least dual role in the initiation and progression of malignant diseases. While the immune system is critical for preventing and/or fighting cancer via processes referred to as immune surveillance and anti-tumoral immunity, the persistent and inappropriate activation of the immune system manifested as (chronic) inflammation has been identified as potent promoter and enabler of malignant development [80, 81]. The persistent production of pro-inflammatory cytokines such as IL6, tumor necrosis factor (TNF) and IL1 within the tumor and its microenvironment plays a key role in mediating the tumor-promoting effect of inflammation (reviewed in [58, 82, 83]).

Several recent studies have provided evidence for reciprocal regulatory interactions of HH/GLI signaling and pro-inflammatory cues during malignant development, including tumor-promoting synergistic signal integration processes. For instance, in pancreatic cancer, stromal HH/GLI signaling has been shown to induce IL6 expression (Fig. 1), which in turn results in paracrine activation of STAT3 in the tumor cell compartment, thereby supporting cancer growth and survival [84]. Another study of pancreatic cancer provided evidence for HH/GLI activation in response to inflammatory TNF and IL1 signaling. Mechanistically, the activation of NFκB by pro-inflammatory signals can induce the expression of GLI1 in a HH-dependent and non-canonical, HH/SMO-independent manner [85]. Similarly, Nakashima et al. investigated the interplay of NFκB and HH pathway activation in human pancreatic cancer, linking IL1, TNF and LPS mediated induction of NFκB signaling with elevated SHH levels and accelerated cancer cell proliferation (Fig. 1) [86].

In BCC, the interaction of HH/GLI and pro-inflammatory IL6/signal transducer and activator of transcription-3 (STAT3) signaling synergistically regulates common GLI-STAT3 target genes and promotes cancer proliferation (Fig. 1) [87, 88]. Furthermore, aberrant regulation of HH/GLI signaling has been implicated in *Helicobacter* induced stomach cancer, where GLI1 function in myeloid cells recruited to the metaplastic area is required for a pro-inflammatory signaling network including IL1 in the myeloid and as a consequence IL6/STAT3 expression in the epithelial compartment [50].

**Fig. 2:**
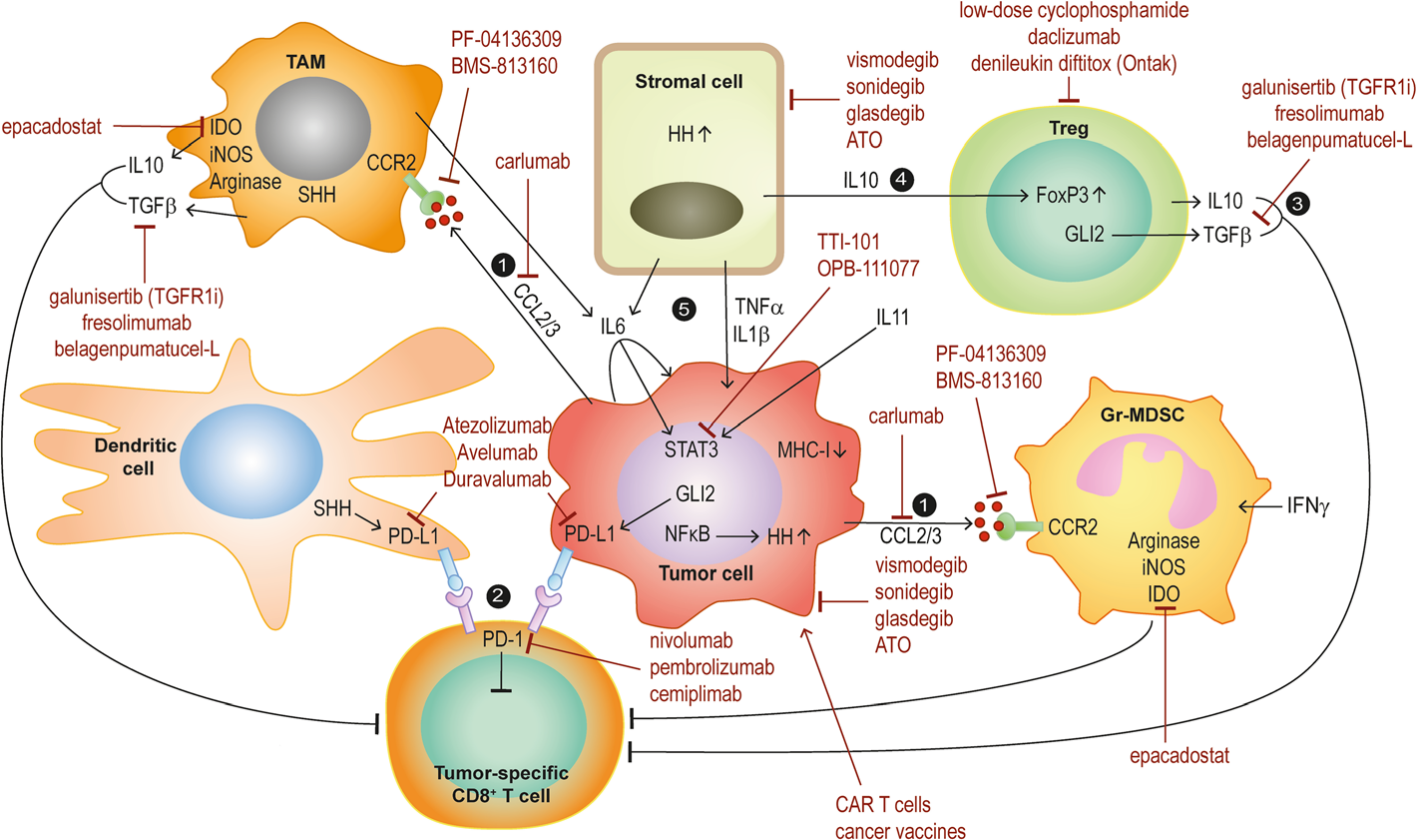
Possible rational combination treatments for HH/GLI-associated cancers simultaneously targeting HH/GLI and HH-regulated immunosuppressive and tumor-promoting signals. The efficacy, response rate and durability of the therapeutic effect of HH/GLI pathway inhibition is likely to increase upon combination of approved HH/SMO antagonists with small molecules and/or biologics such as neutralizing or blocking antibodies developed for the selective inhibition of immune suppression and tumor-promoting inflammation

These findings altogether suggest an intricate interplay of HH/GLI signaling and pro-inflammatory effectors generating a tumor-promoting environment and it will be pivotal to decipher these reciprocal interactions in a cancer entity- and context-dependent manner for the development of future combination therapies interfering with HH/GLI itself together with cooperative pro-inflammatory pathways (Fig. 2).

## Conclusions

Although the field of tumor immunology in the context of oncogenic HH/GLI signaling is relatively young, it has already become evident that HH/GLI signaling exerts complex and diverse effects on the immune microenvironment of malignant and non-malignant tissues. Dysregulation of HH/GLI signaling plays fundamental yet distinct roles both in cancer and chronic inflammatory diseases. For instance, in colitis or pancreatitis, lack of HH expression has been shown to foster chronic inflammation, which is likely to promote tumor formation [45, 49, 52, 69]. By contrast, in several cancer entities, aberrantly activated HH/GLI signaling drives tumor proliferation and growth, while simultaneously dampening inflammation and favoring immunosuppression [53, 54, 64]. Understanding the molecular rationale of how deregulation of the HH/GLI signaling axis precisely alters anti-tumor immunity and tumor-promoting inflammation will support the development of more sophisticated tumor therapies.

Given the immunosuppressive function of HH/GLI, HH antagonists may synergize with immune checkpoint blockers such as anti-PD-1 antibodies in fighting cancer. Notably, single case studies with BCC patients receiving nivolumab or pembrolizumab (two clinically approved anti-PD-1 antibodies) have already yielded promising results, suggesting that the use of immune checkpoint inhibitors can provide a therapeutic benefit in HH/GLI-driven non-melanoma skin cancer [74, 76, 89, 90]. The outcome of recent and ongoing clinical trials with immune checkpoint inhibitors for the treatment of metastatic or unresectable BCC alone or in combination with HH/SMO inhibitors will inform about whether immunotherapy or combinatorial treatments can increase the efficacy and durability of the response of BCC patients (see https://www.clinicaltrials.gov/ trials identifiers: NCT03132636; NCT03521830; NCT02690948). The results of these trials will also have important consequences for the treatment of other HH-associated cancer entities [85, 91].

The patients´ response to immune checkpoint inhibitors correlates with the tumor mutational burden [92]. Given the extraordinary high mutation rate of BCC [62], chances are high that rational combination treatments involving HH pathway inhibitors together with immunotherapeutics (summarized in Fig. 2) will increase the efficacy of current medical therapies of unresectable advanced and metastatic BCC, and possibly also of other HH-associated malignancies with high medical need. In this context it is noteworthy that the immune-modulatory drug imiquimod is already successfully used for the treatment of superficial BCC [58, 93] by boosting T-cell effector function, although there are other reports suggesting an additional therapeutic role of imiquimod such as by directly blunting oncogenic HH/GLI via activation of adenosine receptor/protein kinase A (PKA) signaling and by activating tumor-killing plasmacytoid dendritic cells [94–97].

Despite the promising outlook for the use of HH pathway inhibitors in combination with immunotherapy, there are also challenges and concerns for the use of HH inhibitors as immune modulators. For instance, a study of de la Roche and colleagues has unraveled a role of SMO in the immunological synapse during T-cell activation. Administration of SMO inhibitors led to the functional disruption of the immunological synapse and consequently, to the loss of T-cell effector activity [98]. Although it is unclear whether the administration of SMO inhibitors impedes cytotoxic T-cell functions in patients - which could to some extent explain the failure of several clinical trials with SMO inhibitors [57, 99] - the possible negative impact of HH targeting on the anti-tumoral response needs to be considered in future studies, particularly in those that involve immune checkpoint inhibitors. A better understanding of the effect of HH/GLI pathway modulators and cancer drugs on the immune response is therefore pivotal and will pave the way towards the next generation of combination therapies involving HH/GLI inhibitors and immunotherapeutic drugs.

## Data Availability

Not applicable.

## Abbreviations

AML: Acute myeloid leukemia
AP-1: Activator protein-1
BCC: Basal cell carcinoma
CCL2: C-C motif chemokine ligand 2
CLL: Chronic lymphocytic leukemia
CML: Chronic myeloid leukemia
DLBCL: Diffuse large B-cell lymphoma
GLI: Glioma-associated oncogene
HH: Hedgehog
IFNγ: Interferon gamma
IHH: Indian Hedgehog
IL1: Interleukin-1
IL10: Interleukin-10
IL6: Interleukin-6
MAPK: Mitogen activated protein kinase
MDSC: Myeloid derived suppressor cell
NFκB: Nuclear factor kappa B
PD-1: Programmed death-1
PD-L1: Programmed death ligand-1
PI3K: Phosphoinositide 3 kinase
PKC: Protein kinase C
PTCH1: Patched-1
SHH: Sonic Hedgehog
SLFN4: Schlafen-4
SMO: Smoothened
STAT3: Signal transducer and activator of transcription 3
SUFU: Suppressor of Fused
TAM: Tumor associated macrophages
TNF: Tumor necrosis factor
Treg: Regulatory T-cells
